# Identification of shipping signals with few-shot learning: A distribution-aware approach

**DOI:** 10.1371/journal.pone.0352683

**Published:** 2026-07-09

**Authors:** Bum-Kyu Kim, Sungho Cho, Sunhyo Kim, Hansoo Kim, Ye-Ji Jin, Hyun-Tae Choi, Won-Du Chang

**Affiliations:** 1 Department of Artificial Intelligence Convergence, Pukyong National University, Busan, Korea; 2 Department of Ocean Science, University of Science & Technology (UST), Busan, Korea; 3 Sea Power Reinforcement· Security Research Department, Korea Institute of Ocean Science & Technology, Busan, Korea; Guangdong University of Petrochemical Technology, CHINA

## Abstract

Effective identification of shipping signals in underwater environments is essential for maritime operations and ecosystem monitoring. Traditional models require extensive data for each ship type, posing a significant challenge owing to the difficulty of collecting diverse signals, particularly for vessels with security constraints. Few-shot learning offers a promising solution by enabling ships identification from minimal data through accurate template matching. This study proposes a novel few-shot learning approach that leverages stochastic information within and between ship types to improve identification accuracy using limited labeled data. The proposed model is designed based on a Siamese prototype network that integrates intra- and inter-category dissimilarities, employing cosine distance to estimate similarity while accounting for variance within the data. It achieves robust performance even when trained on limited samples with an average accuracy of 87.81% in five-way identification. In addition, its ability to generalize to unseen ship classes highlights its potential for real-time marine applications, further confirming the effectiveness of few-shot learning in constrained data scenarios. This approach provides valuable insights into designing adaptive, efficient systems for underwater signal detection and has potential applications across a wide range of acoustic processing tasks.

## Introduction

Underwater noise in marine environments originates from various sources, broadly categorized as natural phenomena and human activities [1–3]. In shallow-water zones, natural sources include wind-driven surface noise and sounds produced by marine organisms, whereas human-induced noise primarily comprises those from shipping activities, offshore construction, geological surveys, and sonar operations [3–8]. A significant portion of this anthropogenic underwater noise stems from commercial shipping. The trend toward larger, faster vessels has intensified noise levels, leading to acoustic saturation that adversely affects marine ecosystems [1,9]. To mitigate this impact, the International Maritime Organization (IMO) has been actively considering underwater noise regulations for ships within the Marine Environment Protection Committee since 2008. In 2014, the IMO issued guidelines to reduce noise from commercial ships, further defining international regulatory standards [9].

Following the enactment of IMO regulations, nations have been expected to manage ship traffic selectively within their territorial waters by continuously monitoring underwater noise levels. However, analyzing and detecting shipping signals requires specialized acoustic expertise and advanced analytical capabilities [10]. Recent improvements in acoustic equipment accuracy and the rise of long-term monitoring have led to an accumulation of substantial underwater noise data, increasing the need for automated, data-driven detection methods [11].

Spectrogram analysis is a common method for analyzing underwater acoustic data, visualizing sound in terms of time and frequency [12]. This technique enables the visual identification of the acoustic characteristics of underwater noise sources. For instance, the noise from a moving ship in a spectrogram exhibits a prominent *U*-shaped interference pattern in the 100–1,000 Hz range, reflecting temporal changes [6,7,11]. These *U*-shaped patterns are induced by wave interference across multiple propagation paths—direct, surface-reflected, and seabed-reflected waves—within the waveguide bound by the seabed and sea surface, particularly when broadband sources travel near the surface layer [11,13]. This interference pattern reveals a ship’s presence and movement direction, with descending patterns indicating an approaching ship and ascending patterns a receding one [12–14].

Deep-learning (DL) methods have increasingly been employed in recent decades to automate feature extraction and enhance the accuracy of underwater shipping signal recognition. Doan et al. [15] employed DenseNet to classify various underwater noise sources under low signal-to-noise ratio conditions, whereas Shen et al. [16,17] extracted acoustic features using auditory filter banks and classified shipping signals via convolutional neural networks (CNNs). Hu et al. [18] focused on detecting underwater noise sources using CNNs, and Irfan et al. [19] applied a CNN-based autoencoder to classify shipping signals across four categories: cargo ships, passenger ships, tankers, and tugboats. Yuan et al. [20] leveraged multimodal DL techniques, using both shipping signal and visual imagery for signal detection.

Moreover, public datasets such as DeepShip [19], ShipsEar [21], and QiandaoEar22 [22] provide complementary scales, metadata, and channel configurations, supporting reproducible benchmarking across varied real-world conditions (season/sea-state variability, multi-target scenes, multi-channel sensing). DeepShip comprises ship‐radiated noise recorded in real seas for approximately 47 h 4 min spanning four classes. ShipsEar is an in-situ database of ship‐radiated noise that includes 11 vessel categories. QiandaoEar22 targets multi-target scenarios and contains 9 h 28 min of ship-noise recordings together with 21 h 58 min of background noise. Despite these advances, applying these methods in real-world shipping-signal recognition remains challenging owing to the difficulty of collecting datasets comprising a diverse range of signals. Most autonomous models require extensive datasets for each ship category to achieve accurate identification performance. However, obtaining signals from certain types of ships can be particularly difficult, often owing to security constraints, resulting in data scarcity for key categories. Few-shot learning offers a solution by enabling the identification of previously unseen classes using only a few examples per class [23]. It trains models to measure similarity or dissimilarity between query and key signals, enabling accurate prediction of signal classes from unseen categories, even with limited query signals from new categories.

This study proposes a novel few-shot learning approach to distinguish various underwater shipping signals without introducing additional learnable parameters. This approach leverages stochastic information within and between ship types to enhance identification accuracy with limited labeled data. By applying self-calibration (SC), inter-calibration (IC), and boundary loss (BL) to the support and query sets, the prototype computation is refined to enhance classification performance. In addition, cosine distance is employed to effectively capture similarities between data, enabling accurate classification even for previously unseen ship signals.

## Related work

**Fig 1:**
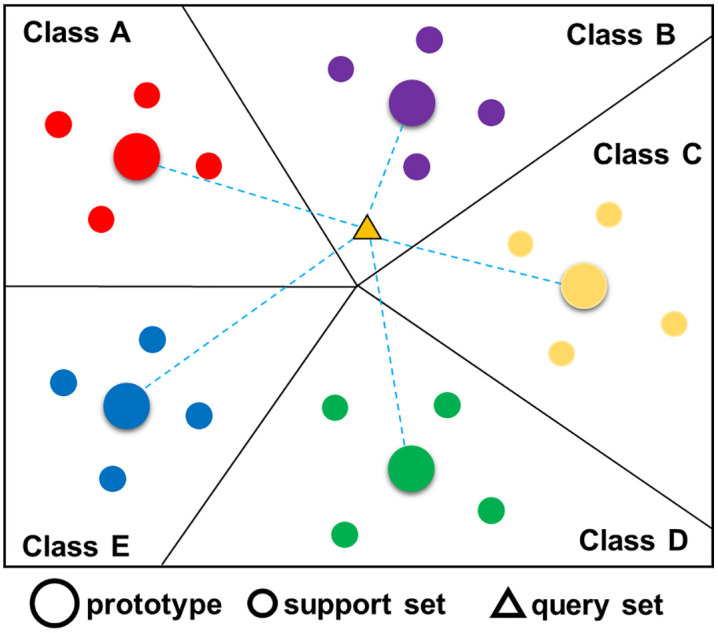
Prototypical networks in few-shot learning scenarios. Prototypes (class means) are calculated from support-set embeddings, and query examples are classified based on their distance to these prototypes.

Few-shot learning addresses the challenge of classifying previously unseen classes with limited training examples by learning a generalized approach to identify classes [24–26]. These methods are broadly categorized into model-, metric-, and optimization-based approaches. Among these, the metric-based approach has proven particularly effective for classifying new classes with limited data by measuring the distance between data points [27]. Fig 1 illustrates the prototypical concept of metric-based few-shot learning. In this approach, the model learns to calculate the dissimilarity between the mean of the support signals (prototypes) and each query signal to identify the class with the highest probability. When a new type of signal appears—such as class F—the model can readily accommodate it by simply updating the prototypes with a few examples from the new class (e.g., class C).

### Among conventional metric-based methods, the prototypical network

(ProtoNet) proposed by Snell et al. [23] stands out for its simplicity and effectiveness, inspiring ongoing research [28–31]. Building upon these advancements, the Siamese prototype network (SPNet) was proposed to enhance the precision of few-shot learning by preserving similarity within the embedding space and achieving high classification accuracy even with limited data [32]. These studies have introduced methods to refine prototypes using mechanisms such as attention, leading to improvements in model accuracy, adaptability, and interpretability in complex classification tasks. These modifications contribute to performance gains, with a focus on efficiency, and are particularly useful in resource-constrained environments. Furthermore, they extend the applicability of few-shot learning to applications requiring rapid model adaptation with minimal data.

Several studies have leveraged metric-based few-shot learning to detect and classify shipping signals. Tian et al. [33] and Xie et al. [34] employed cosine similarity within a few-shot learning framework to identify underwater noise sources. Similarly, Nie et al. [35] employed contrastive learning based on Euclidean distance for underwater noise classification. In addition, various other studies have demonstrated the effectiveness of few-shot learning for underwater noise classification [36–41].

In contrast to previous studies, this work focuses on enhancing shipping-signal identification by leveraging stochastic information both within and between ship types. This study adopts cosine distance to achieve more robust similarity estimation. Furthermore, by applying SC and IC to the support and query sets, along with incorporating BL, the prototype representations are refined, resulting in improved classification performance.

## Materials and methods

### Data and preprocessing

**Fig 2:**
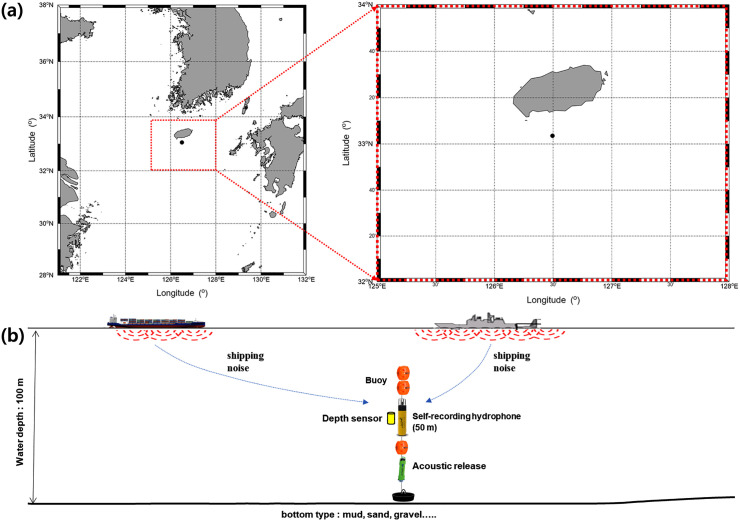
Study area and acoustic-monitoring setup (a) location of self-recording hydrophone in Jeju Island’s southern sea; (b) experimental layout for recording ship-radiated signals. This map was generated using the M_Map mapping package for MATLAB [43]. Coastline and boundary data were derived from the Global Self-consistent, Hierarchical, High-resolution Geography (GSHHG) database [44], which is licensed under the GNU Lesser General Public License.

This study was conducted using underwater acoustic data recorded in the southern sea of Jeju Island [1,2]. This area is characterized by heavy ship traffic from large cargo ships and tankers traversing the East China Sea, providing a rich dataset for studying underwater acoustic signals. The region is also rich in fish resources, supporting active fishing and regular operations of cruise and passenger ships, contributing to a diverse soundscape [4,8,42]. The underwater acoustic data used in this study were collected continuously in this region from July 15–26, 2016 (Fig 2A). All acoustic measurements were conducted in open coastal waters where no specific research permits were required, as these data were acquired in non-restricted marine areas [1,2,4]. These signals were recorded using a self-recording hydrophone deployed at a depth of approximately 50 m and located about 18 km south of Jeju Island (Fig 2B). Stable, long-term data acquisition was ensured by using an underwater buoy for mid-water mooring, minimizing the risk of equipment loss owing to potential ship collisions, which is a concern with surface buoys. The hydrophone was configured with a sampling frequency of 24 kHz and a system gain of 12 dB to ensure adequate signal amplification. A high-pass filter with a cutoff frequency of 40 Hz was applied to reduce low-frequency noise in the acoustic data.

The frequency components of the recorded broadband time-series data were analyzed using the short-time Fourier transform. This method involves performing a fast Fourier transform (FFT) on consecutive overlapping segments of the acoustic signal, effectively generating a spectrogram that provides a time-frequency representation. The Fourier transform decomposes the input signal into a sum of periodic functions with various frequencies, enabling detailed analysis of these components. Spectrograms, with their time–frequency representation, facilitate the analysis of acoustic characteristics from various sources, both above and underwater, rendering them a fundamental tool for source analysis in underwater acoustics.

The underwater acoustic data recorded during the observation period were saved as individual WAV files every 10 min, resulting in 1,572 audio files. Acoustic signals from ships passing within a 5 km radius of the hydrophone were extracted and segmented into 1-s intervals for training and validation (Table 1). To accurately identify passing ships and associate with their acoustic signatures, dynamic and static information, such as location, speed, and direction, was obtained in real-time from the Automatic Identification System (AIS). This study used Maritime Mobile Service Identity (MMSI) as a unique identifier for each ship to recognize it individually. In contrast, the ship type number is a unique classification code assigned by AIS, and is used to distinguish ship categories such as large cargo ships and passenger vessels. A total of 21 ships passed within the 5 km radius during the study period. Spectrograms were constructed from the acoustic signals of these ships.

**Table 1:** Information and passage times of ships crossing within 5 km of the hydrophone.

Number	MMSI	Ship type	Ship type number	Start time	End time
1	351034000	cargo	70	2016-07-16 14:27:22	2016-07-16 14:42:40
2	440043000	cargo	70	2016-07-17 03:31:27	2016-07-17 03:44:37
3	440090000	cargo	70	2016-07-19 11:58:30	2016-07-19 12:22:51
4	440629000	cargo	70	2016-07-15 21:06:22	2016-07-15 21:39:41
5	671645000	cargo	70	2016-07-16 01:51:28	2016-07-16 02:10:09
6	354434000	tanker	81	2016-07-16 08:57:51	2016-07-16 09:22:41
7	357708000	tanker	83	2016-07-17 23:24:30	2016-07-17 23:47:01
8	373933000	tanker	80	2016-07-18 03:26:35	2016-07-18 03:50:24
9	413439970	tanker	80	2016-07-20 11:14:44	2016-07-20 11:44:54
10	440207000	tanker	80	2016-07-16 00:14:24	2016-07-16 00:38:03
11	440470000	tanker	89	2016-07-18 15:40:11	2016-07-18 16:00:36
12	440978000	tanker	81	2016-07-18 01:00:08	2016-07-18 01:21:08
13	441151000	tanker	80	2016-07-16 23:02:39	2016-07-16 23:30:39
14	441269000	tanker	70	2016-07-18 11:44:25	2016-07-18 12:12:23
15	441361000	tanker	80	2016-07-20 10:31:01	2016-07-20 10:55:31
16	441555000	tanker	82	2016-07-17 12:30:37	2016-07-17 12:55:27
17	441578000	tanker	80	2016-07-20 13:07:50	2016-07-20 13:48:20
18	441659000	tanker	81	2016-07-22 07:26:43	2016-07-22 07:26:49
19	441805000	container	70	2016-07-19 23:47:15	2016-07-20 00:08:39
20	351841000	container	79	2016-07-16 16:31:49	2016-07-16 16:42:25
21	351577000	container	70	2016-07-21 09:09:04	2016-07-21 09:26:04

**Fig 3:**
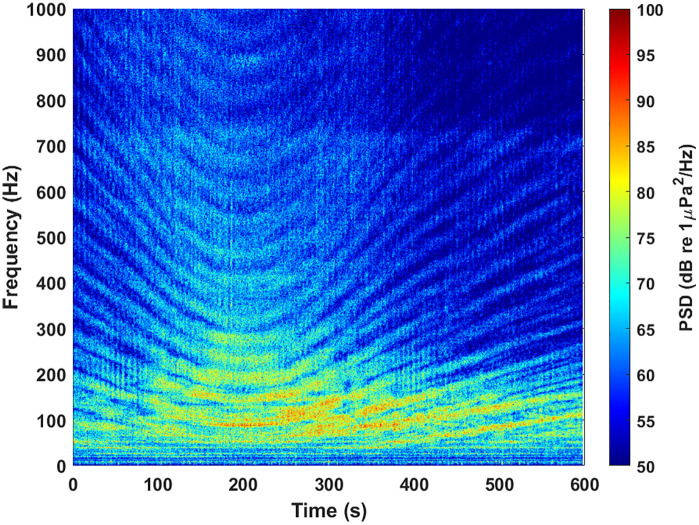
Spectrogram of a shipping signal exhibiting the characteristic *U*-shape interference pattern.

Each spectrogram was generated by processing the corresponding acoustic signal using the FFT to calculate the dominant frequency components. The primary frequency range of shipping signals, where the characteristic *U*-shaped interference pattern prominently appears, was determined to be between 100 and 1000 Hz (Fig 3). Subsequently, individual datasets for each ship’s acoustic signal were constructed at 1-s intervals. These spectrograms were resized to (224, 224, 3) images for compatibility with the selected DL model and normalized to values between zero and one to enhance training efficiency. In addition, for data augmentation during training, Gaussian noise with a standard deviation of 0.02 was applied to the normalized input images before model input.

### Proposed method

This study proposes a method based on the Prototypical Network [23], a metric-based few-shot learning model that performs classification by calculating the distance between the embedded feature vector of a test sample and each prototype. To rigorously evaluate the ability to classify unseen classes that were not encountered during training, this study followed a meta-learning protocol. The dataset was divided into two groups: meta-training and meta-test, which comprise 16 and 5 ship classes, respectively. The meta-training set was used for episodic training, whereas the meta-test set was reserved exclusively for the final evaluation of generalization performance. The evaluation was conducted exclusively using the five unseen classes in the meta-test set with an *n*-way, *n*-shot episodic scheme. Given the limitation of having only 21 available ship classes, a separate Meta-validation set was intentionally omitted to maximize the use of available classes for meta-training and meta-test.

Meta-training was performed using an episodic learning approach composed of multiple training episodes. In each episode, *C* classes were randomly selected and *K* samples were then randomly drawn from each class to construct a *C* × *K* support set. Subsequently, a query set was constructed by randomly sampling from the same *C* classes. The model was then trained to classify the query samples among the C classes by minimizing the distance to the target class while maximizing the distance to the other classes. This setting is referred to as a *C*-way, *K*-shot problem, as it utilizes K samples per class in the support set. As training proceeds over episodes, the model learns commonalities across meta-tasks, such as extracting important features and comparing sample similarities, while disregarding task-specific nuances. This learning mechanism enables the trained model to generalize effectively to new, unseen meta-tasks. The entire process of data splitting and episodic learning was repeated 50 times to evaluate the generalizability of the proposed method.

**Fig 4:**
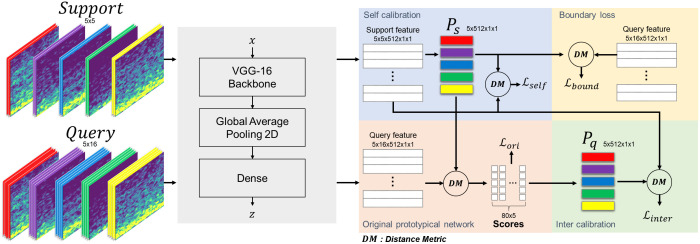
Architecture of the proposed network for five-way, five-shot classification.

Fig 4 presents the overall workflow of the proposed framework under a five-way, five-shot setting. In each episode, five support samples are selected from each of the five classes, and 16 query samples per class are simultaneously provided, corresponding to the 5 × 5 support features and 5 × 16 query features shown in the figure. The support and query spectrograms are passed through a shared VGG-16 backbone, followed by a global average pooling layer and a dense layer, to produce fixed-length embedded feature vectors. These vector representations are then used to compute the loss terms for training. Specifically, the framework adopts the original, self-calibration, and inter-calibration losses from previous studies [23,32] and further introduces boundary-calibration loss to penalize query samples that intrude into the boundary regions of non-target classes. The final objective function is formulated as a weighted combination of these loss terms. In addition, a hard-negative strategy was proposed alongside standard softmax for the conventional losses in previous studies [23,32]. Unlike standard softmax, which incorporates all class scores in the loss calculation, the proposed strategy constructs the loss by contrasting the target class with the most competitive non-target class.

Self-calibration loss (SCL) [32] is computed by measuring the distances between the embedded vectors of the support set S and the class prototype $\overset{c}{\underset{s}{P}}$, which is defined as the mean vector of the embedded support samples in class c. It encourages support samples to remain close to their own class prototype while being distinguished from other class prototypes. The SCL for the support set is defined as follows:


$$\begin{array}{c} L_{self}=\;-\frac{\text{1}}{C\cdot K}\sum_{i=\text{1}}^{C\cdot K}\text{log}p\left(y_{i}|\overset{s}{\underset{i}{z}}\right)\;, \end{array}$$ (1)

$$\begin{array}{c} p\left(y_{i}=c|\overset{s}{\underset{i}{z}}\right)=\frac{exp\left(u_{ic}\right)}{\sum_{c^{\prime }}^{C}exp\left(u_{ic^{\prime }}\right)}, \end{array}$$ (2)

$$\begin{array}{c} u_{ic}=-d\left(\overset{s}{\underset{i}{z}},\;\overset{s}{\underset{c}{P}}\right), \end{array}$$ (3)

where $\overset{s}{\underset{i}{z}}$ denotes the embedded vector of the $i^{th}$ sample in the support set S, $y_{i}$ the label of the sample, and $\;\overset{s}{\underset{c}{P}}$ the mean vector of the embedded support samples in class c.

Original classification loss (OCL) [23] is designed to encourage each query sample to be associated with its target support-set prototype $\overset{s}{\underset{c}{P}}$ in the embedding space. Based on the distance between an embedded query vector and each support-set prototype, class-wise scores are obtained for each query sample. The OCL is then defined from the probability assigned to the target class, formulated as follows:


$$\begin{array}{c} L_{ori}=\;-\frac{\text{1}}{N_{q}}\sum_{i=\text{1}}^{N_{q}}\text{log}p\left(y_{i}|\overset{q}{\underset{i}{z}}\right), \end{array}$$ (4)

$$\begin{array}{c} p\left(y_{i}=c|\overset{q}{\underset{i}{z}}\right)=\frac{exp\left(u_{ic}\right)}{\sum_{c^{\prime }}^{C}exp\left(u_{ic^{\prime }}\right)}, \end{array}$$ (5)

$$\begin{array}{c} u_{ic}=-d\left(\overset{q}{\underset{i}{z}},\;\overset{s}{\underset{c}{P}}\right), \end{array}$$ (6)

where $\overset{q}{\underset{i}{z}}$ denotes the embedded vector of the $i^{th}$ sample in the query set Q, $N_{q}$ the total number of samples in the query set Q, and $y_{i}$ the label of the sample as defined in Eq. (3).

Inter-calibration loss (ICL) [32] is designed to encourage each support sample to be associated with its corresponding query-set prototype $\overset{q}{\underset{c}{P}}$, thereby reversing the direction of the prototype assignment. Based on the distance between an embedded support vector and each query-set prototype, class-wise scores are obtained for each support sample. The ICL is then defined as follows:


$$\begin{array}{c} L_{inter}=\;-\frac{\text{1}}{C\cdot K}\sum_{i=\text{1}}^{C\cdot K}\text{log}p\left(y_{i}|\overset{s}{\underset{i}{z}}\right), \end{array}$$ (7)

$$\begin{array}{c} p\left(y_{i}=c|\overset{s}{\underset{i}{z}}\right)=\frac{exp\left(u_{ic}\right)}{\sum_{c^{\prime }}^{C}exp\left(u_{ic^{\prime }}\right)}, \end{array}$$ (8)

$$\begin{array}{c} u_{ic}=-d\left(\overset{s}{\underset{i}{z}},\;\overset{q}{\underset{c}{P}}\right), \end{array}$$ (9)

where $\overset{s}{\underset{i}{\text{z}}}$ represents the embedded feature vector of the $i^{th}$ support sample, $y_{i}$ denotes the label of the sample, and $\overset{q}{\underset{c}{P}}$ is the prototype of class c computed from the query set. By minimizing this loss, the model promotes consistency between support representations and query-derived prototypes.

The proposed boundary-calibration loss (BCL) is designed to improve class separation by penalizing query samples that fall within the boundary regions of non-target classes. For each class, the boundary is centered at the corresponding support-set prototype, and its radius is defined as the maximum distance from the prototype to the embedded vectors of the class. Using this boundary, the model measures the intrusion of each query sample into the regions of non-target classes. A penalty is assigned whenever a query sample lies inside any such boundary, and the total intrusion is averaged over all query samples to define the loss. The BCL is formulated as follows:


$$\begin{array}{c} L_{bound}=\;\frac{\text{1}}{N_{q}}\sum_{i=\text{1}}^{N_{q}}\sum_{c\neq y_{i}}I_{ic}\;, \end{array}$$ (10)

$$\begin{array}{c} I_{ic}=\text{max}\left(\text{0},r_{c}-d\left(\overset{q}{\underset{i}{z}},\overset{s}{\underset{c}{P}}\right)\right), \end{array}$$ (11)

$$\begin{array}{c} r_{c}=\underset{\overset{s}{\underset{j}{z}}\in S_{c}}{\text{max}}d\left(\overset{s}{\underset{j}{z}},\overset{s}{\underset{c}{P}}\right)\;, \end{array}$$ (12)

where $\overset{q}{\underset{i}{\text{z}}}$ denotes the embedded vector of the $i^{th}$ query sample, $y_{i}$ the label of that query sample, $\overset{s}{\underset{j}{\text{z}}}$ the embedded vector of the $j^{th}$ support sample, $\overset{s}{\underset{c}{P}}$ the support-set prototype of class c, $S_{c}$ the set of support samples belonging to class c, and $r_{c}$ the class-specific boundary radius.

The proposed hard-negative strategy is designed to focus training on the most competitive non-target class. Unlike standard softmax, which computes the classification loss using the scores of all classes, this strategy constructs a hard-negative logit for each sample by subtracting the highest non-target score from the target-class score. The resulting logit is optimized using binary cross-entropy, encouraging the target-class score to remain larger than that of the hardest negative class. Replacing Eqs. (5) and (8), the hard-negative strategy is defined as follows:


$$\begin{array}{c} p\left(y_{i}=c|z_{i}\right)=\frac{\text{1}}{\text{1}+\text{exp}\left(-\left(\overset{\text{pos}}{\underset{i}{u}}-\overset{\text{neg}}{\underset{i}{u}}\right)\right)}, \end{array}$$ (13)

$$\begin{array}{c} \overset{\text{pos}}{\underset{i}{u}}=\sum_{c=\text{1}}^{C}u_{ic}1\left(c=y_{i}\right), \end{array}$$ (14)

$$\begin{array}{c} \overset{\text{neg}}{\underset{i}{u}}=\underset{c\neq y_{i}}{\text{max}}u_{ic}, \end{array}$$ (15)

where $u_{ic}$ denotes the class-wise score of sample i for class c, $\overset{\text{pos}}{\underset{i}{u}}$ the score assigned to the target class, $\overset{\text{neg}}{\underset{i}{u}}$ the highest score among non-target classes, $y_{i}$ the label of sample i, and 1(·) is the indicator function.

Finally, the overall objective is defined as a linear combination of these four losses as follows:


$$\begin{array}{c} L=\;\alpha L_{ori}+\;\beta L_{SC}+\;\gamma L_{IC}+\;\delta L_{BL}, \end{array}$$ (16)

where 𝛼, 𝛽, γ, and δ are the weights of the four loss components, set to 1, 3, 7, and 0.3 during the experiments, respectively. The values of 𝛼, 𝛽, and γ were adopted as reported in [32], whereas δ was determined empirically. The effects on model performance are further analyzed in the Results section.

## Results and discussion

The models were trained for 700 episodes, as this was empirically found to be the point at which most methods, including the proposed one, reached stable performance. Unless otherwise stated, the experiments were conducted using a 512-dimensional latent vector. Following [32], cosine distance was adopted as the distance metric, and the resulting values were scaled by a factor of 50, as proposed in that study, yielding a range from 0 to 50.

Table 2 presents a comparison between the conventional standard softmax-based classification and the proposed hard-negative strategy. The proposed strategy emphasizes the most competitive non-target class during optimization. As described in the Proposed method section, the difference between the two settings lies in the original, self-calibration, and inter-calibration losses, while the boundary-calibration loss was retained in both settings. Across 50 independent trials, the proposed strategy achieved a mean accuracy of 87.81%, slightly higher than the 87.59% obtained with the standard softmax-based setting. This result indicates that focusing on the hardest negative class can provide a modest performance gain without compromising stability.

**Table 2:** Performance comparison between the conventional softmax-based classification and the proposed hard-negative strategy.

Logit strategy	Mean accuracy (%)	Standard deviation
Hard-negative strategy	87.81	5.94
Standard softmax	87.59	6.33

To evaluate the effect of the proposed BCL, a sensitivity analysis was conducted by varying its weight from 0.0 to 0.5 under a fixed training-time data augmentation of Gaussian noise with a standard deviation of 0.02 (Table 3). The highest mean accuracy, 87.81%, was achieved at a weight of 0.3, corresponding to an improvement of 0.24%p over the baseline without BCL (87.57% at weight 0.0). Although the improvement was modest, the results suggest that an appropriate BCL weight can provide a consistent performance benefit. When the weight exceeded 0.3, accuracy tended to decrease. This finding indicates that a moderate boundary constraint helps improve inter-class discriminability, whereas an excessively large weight may overemphasize the boundary term and weaken the balance with the main classification objective, ultimately reducing generalization performance.

**Table 3:** Sensitivity analysis of the BCL weight on classification performance under the hard-negative strategy and Gaussian noise (σ = 0.02).

Backbone (Latent vector)	Mean accuracy (%)	Standard deviation
0.0	87.57	5.64
0.1	87.68	6.43
0.2	87.55	5.79
0.3	87.81	5.94
0.4	87.69	5.45
0.5	87.32	6.29

To examine the effect of Gaussian noise used as a training-time augmentation, a comparative experiment was conducted by adding Gaussian noise with a standard deviation of 0.02 during training (Table 4). The results showed that the model achieved a mean accuracy of 87.81% when Gaussian noise was used, compared to 87.68% when it was not used, corresponding to an improvement of approximately 0.13%p. Although the gain was modest, the result suggests that Gaussian noise can serve as an effective augmentation method by reducing overfitting to specific training patterns and improving generalization performance.

**Table 4:** Performance comparison according to the presence of Gaussian noise under the hard-negative strategy and boundary-calibration loss (BCL).

Gaussian Noise (0.02)	Mean accuracy (%)	Standard deviation
With	87.81	5.94
Without	87.68	5.81

Table 5 presents the performance of the proposed framework across various backbone architectures under identical experimental conditions without Gaussian noise augmentation. In this comparison, the proposed framework, incorporating the hard-negative strategy and boundary-calibration loss (BCL) with δ=0.3, was implemented with each backbone to evaluate its performance and stability. The results show that the framework achieved competitive performance across all evaluated backbones, with mean accuracies ranging from 87.37% for ViT-B/32 (latent vector size: 768) to 88.07% for VGG-16 (latent vector size 4096). The performance differences were modest across the compared architectures, while standard deviations ranged from 5.81% to 6.58%, indicating generally stable performance. Overall, these results indicate that the proposed framework performs consistently across multiple backbone architectures. However, the observed differences suggest that factors such as representation dimensionality may influence performance, and further validation will be needed to clarify their effects in future work.

**Table 5:** Performance comparison across various backbone architectures under the hard-negative strategy and boundary-calibration loss (BCL), without Gaussian noise augmentation.

Backbone (Latent vector)	Mean Accuracy (%)	Standard Deviation (%)
VGG-16 (4096)	88.07	6.10
ResNet-50 (2048)	88.02	6.12
EfficientNet-B0 (1280)	87.81	5.95
ResNet-18 (512)	87.80	6.10
Inception-V3 (2048)	87.76	5.90
VGG-16 (512)	87.68	5.81
ViT-B/32 (768)	87.37	6.58

Table 6 presents the classification accuracies of the proposed network in comparison with representative few-shot learning methods. To support a fairer comparison, the primary comparison was conducted without Gaussian noise augmentation for all methods, while the Gaussian-noise-augmented version of the proposed network was additionally reported as a reference. Methods marked with “†” use the same VGG-16 backbone as our method to reduce the influence of backbone differences, whereas the unmarked methods follow their original backbone configurations. A backbone-controlled setting was not additionally introduced for MAML and MatchingNet because their original formulations differ more substantially from the prototype-based methods in terms of learning and inference procedures.

**Table 6:** Classification comparison of the proposed network with representative few-shot learning methods.

Method	Gaussian Noise (GN)	Mean Accuracy (%)	Std. Dev. (%)
Proposed network (Baseline)	Without	87.68	5.81
Proposed network	With	87.81	5.94
SPNet [32] ^†^	Without	87.24	6.78
ProtoNet [23] ^†^	Without	87.11	6.00
SPNet [32]	Without	84.65	7.48
ProtoNet [23]	Without	84.49	7.05
MAML*	Without	79.46	9.04
MatchingNet *	Without	74.88	9.97
RelationNet ^†^	Without	68.82	12.20
RelationNet	Without	67.88	11.35

^†^ Methods with “†” use the same VGG-16 backbone as our method, whereas the unmarked methods use their original backbone configurations.

* A backbone-controlled setting was not additionally introduced for MAML and MatchingNet because their original formulations differ more substantially from the prototype-based methods in terms of learning and inference procedures.

The baseline of the proposed network achieved the highest mean accuracy (87.68%) with a modest margin over the compared methods. Under the original backbone settings, it outperformed SPNet, ProtoNet, MAML, MatchingNet, and RelationNet [45]. Under the backbone-controlled setting, it also achieved higher accuracy than SPNet† and ProtoNet†. Notably, SPNet† corresponds to a setting in which both the hard-negative strategy and boundary-calibration loss (BCL) are absent. As shown in Tables 2 and 3, the hard-negative strategy and BCL yielded improvements of 0.22%p and 0.24%p, respectively. Consistent with these findings, Table 6 shows that the proposed method achieved an accuracy improvement of 0.44%p when both the hard-negative strategy and boundary-calibration loss (BCL) were incorporated, and the additional use of Gaussian noise augmentation increased the total gain to 0.57%p.

Taken together, the results suggest that the proposed components, designed to make use of data-distribution-related information, contributed to the observed performance improvements. The hard-negative strategy, boundary-calibration loss (BCL), and Gaussian noise augmentation each showed positive effects, and their combined use produced the best overall performance. At the same time, the total improvement remained limited to approximately 0.57%p, indicating that further studies on backbone selection and parameter optimization are still needed. Nevertheless, under the backbone-controlled setting, the proposed method appears to provide a more effective extension of the original ProtoNet framework for underwater acoustic data than SPNet. For reference, the performance difference between ProtoNet and SPNet was 0.13%p, with SPNet incorporating two additional loss terms over ProtoNet. It should also be noted that the present experiments were conducted using only underwater acoustic data, and further validation using a wider variety of datasets will be needed for a more comprehensive comparison.

## Conclusions

This study investigated a Siamese network-based few-shot learning approach for the identification of underwater acoustic shipping signals, achieving robust performance in data-scarce scenarios. By integrating domain-specific constraints—specifically the hard-negative softmax and BCL—the proposed framework effectively structured the embedding space to enhance inter-class discriminability. Experimental results confirmed that the proposed network achieved a peak mean accuracy of 87.81% in a five-way, five-shot setup, significantly outperforming traditional few-shot learning methods such as MAML, MatchingNet, and RelationNet. At the same time, the improvement over ProtoNet and SPNet was relatively modest, indicating a limited performance margin over closely related prototype-based methods.

A major highlight of this work is the systematic cross-backbone comparison, which revealed that a moderately complex VGG-16 architecture with a 512-dimensional latent vector provides superior stability and reliability, recording the lowest standard deviation (5.81%) among various state-of-the-art architectures. This demonstrates that the integration of boundary-aware loss functions with a stable, lightweight architecture provides a reliable solution for practical maritime surveillance. Despite these achievements, environmental variables such as water temperature and bathymetry were not explicitly modeled in this study. Future research should aim to extend this framework to multi-vessel environments and investigate self-supervised adaptation techniques to further enhance generalizability across diverse oceanic regions. Nevertheless, given its high accuracy and computational efficiency, the proposed model is well-suited for real-time deployment in autonomous maritime monitoring and embedded surveillance systems.

## Supporting information

S1 File Shipping_signal. (7Z)
